# The cognitive-interpersonal maintenance model of anorexia nervosa revisited: a summary of the evidence for cognitive, socio-emotional and interpersonal predisposing and perpetuating factors

**DOI:** 10.1186/2050-2974-1-13

**Published:** 2013-04-15

**Authors:** Janet Treasure, Ulrike Schmidt

**Affiliations:** 1 Department of Psychological Medicine, King’s College London, Institute of Psychiatry, The Basement, P059, 103 Denmark Hill, London, SE5 8AF, UK

**Keywords:** Anorexia nervosa, Model, Complex intervention, Eating disorder

## Abstract

**Aim:**

To describe the evidence base relating to the Cognitive-Interpersonal Maintenance Model for anorexia nervosa (AN).

**Background:**

A Cognitive-Interpersonal Maintenance Model maintenance model for anorexia nervosa was described in 2006. This model proposed that cognitive, socio-emotional and interpersonal elements acted together to both cause and maintain eating disorders.

**Method:**

A review of the empirical literature relating to the key constructs of the model (cognitive, socio-emotional, interpersonal) risk and maintaining factors for anorexia nervosa was conducted.

**Results:**

Set shifting and weak central coherence (associated with obsessive compulsive traits) have been widely studied. There is some evidence to suggest that a strong eye for detail and weak set shifting are inherited vulnerabilities to AN. Set shifting and global integration are impaired in the ill state and contribute to weak central coherence. In addition, there are wide-ranging impairments in socio-emotional processing including: an automatic bias in attention towards critical and domineering faces and away from compassionate faces; impaired signalling of, interpretation and regulation of emotions. Difficulties in social cognition may in part be a consequence of starvation but inherited vulnerabilities may also contribute to these traits. The shared familial traits may accentuate family members’ tendency to react to the frustrating and frightening symptoms of AN with high expressed emotion (criticism, hostility, overprotection), and inadvertently perpetuate the problem.

**Conclusion:**

The cognitive interpersonal model is supported by accumulating evidence. The model is complex in that cognitive and socio-emotional factors both predispose to the illness and are exaggerated in the ill state. Furthermore, some of the traits are inherited vulnerabilities and are present in family members. The clinical formulations from the model are described as are new possibilities for targeted treatment.

## Background

Treatment for anorexia nervosa (AN) has focused on remediating the eating and weight symptoms. Historically, inpatient care was used but the NICE guidelines recommended outpatient care in the first instance. In the early phase of adolescent AN, mobilising the parents to restore a normal eating pattern is effective [1-3]. Nevertheless a sizeable proportion of patients (30-60%) fail to change their behaviour. In adult patients, particularly if the duration of illness has lasted over 3 years, change is more difficult [4].

Premorbid vulnerabilities contribute to a poor response to treatment. Cognitive rigidity and obsessive compulsive traits [5] and social communication difficulties are associated with a poorer outcome from AN [6]. Furthermore, secondary consequences of malnutrition that accumulate over time contribute to treatment resistance. A starved brain, for instance, is less plastic and hence responds less well to talking treatments which require changes to be made in reflective cognitive functions.

Features such as these led us to develop a cognitive-interpersonal maintenance model for AN. The essential features of this model were that there were predisposing factors such as obsessive compulsive features and anxious avoidance (particularly of close relationships) increased the vulnerability to AN and that these contributed also to the maintenance of the disorder because they fostered pro anorexia nervosa beliefs and behaviours. These traits cause problems relating to others and, in addition, the highly visible eating disorders (ED) symptoms and behaviors have a profound effect on other people and lead them to react in ways which in turn serve to maintain the disorder. Thus, interpersonal relationships become entangled with the disorder in a complex manner.

The predictions from this model were that obsessive compulsive and anxious avoidant traits would be intermediate phenotypes increasing the risk of the disorder developing and that social processing and interpersonal interactions might be problematic. It follows that treatment for AN would therefore need to be a complex form of intervention with multiple interacting components. The Medical Research Council (MRC) has developed guidelines on the development of complex interventions as it is acknowledged that they entail unique challenges [7]. A key step is to develop a strong theoretical understanding in order to target the processes and outcomes that are integral to recovery. Developing the theoretical framework is an ongoing process and the model may need to be adjusted as new evidence emerges.

The aim of this paper is to examine the evidence relating to the cognitive interpersonal model. First, we scrutinise and synthesise the literature on genes, molecules, cells, circuits, physiology, behavior and self report measures relating to the cognitive phenotype (OCPD traits) and the anxious, avoidant, social emotional phenotype. We then summarise the evidence relating interpersonal aspects of the illness. Lastly, we describe how clinical formulations and treatments follow from the model.

### Premorbid traits

In the original model, obsessive compulsive traits (OCP) traits were considered to be a key vulnerability factor [8][9] contributing to maintenance through pro anorexia nervosa beliefs and behaviours. Cognitive processing styles such as set shifting and central coherence [10-12] underpin these traits and have been the subject of highly active examination.

### Set shifting

#### Behavioural studies

People with EDs (AN and BN) have moderate to large sized problems with set shifting in a range of tasks (as summarised in a systematic review see [13]). After recovery, these inefficiencies remain in an attenuated form [14,15]. Adolescents with anorexia nervosa are also less impaired [16-19]. This suggests that set shifting may be accentuated as a consequence of the illness. The alternative explanation is that individuals with less pronounced problems in this area are more likely to recover and that these serve as moderating factors. First degree relatives (sisters, twins, parents) of people with eating disorders also perform poorly on some tasks (WCST) [15,20].

**Figure 1:**
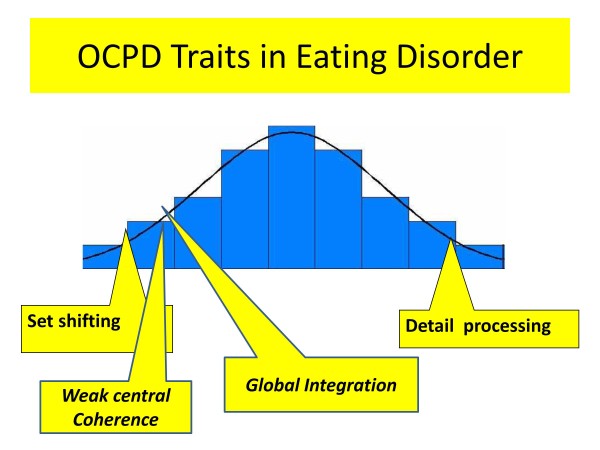
**Obsessive compulsive personality traits.** Legend A diagrammatic representation of traits related to obsessive compulsive personality disorder (OCPD) in eating disorders . Those that are mainly present in the acute, starved state are shown in italics.

Furthermore, in a study by Kothari et al., (2012), children (aged 8) of women with EDs perform poorly on a set shifting task [21]. In summary reduced set shifting is a feature of AN in the acute state. Furthermore, this trait is also present in first degree relatives which suggests that it might be an inherited vulnerability for AN. It is less pronounced after recovery (see Figure 1) suggesting that it is accentuated by starvation.

#### What do we know about the underpinning biology of set shifting?

Set shifting tasks involve increased frontal and parietal and decreased striatal activation in AN [22] and BN [23]. One interpretation is that these tasks may involve more cognitive effort in people with EDs.

Set shifting difficulties are a generic risk factor for other forms of psychopathology. It appears to result from a biological vulnerability in corticostriatal circuits. Set shifting in rodents [24] and in primates is associated with frontal lobe function [25]. An inbred mouse model (BTBR) has problems in set shifting [26,27], Thus, set shifting inefficiencies seen in EDs may be linked to genetic factors that control brain development.

### Central coherence

Weak central coherence (an inability to see the forest for the trees) is thought to arise from an imbalance between global and detail processing and is common in people with EDs. People with EDs have a moderate to large superiority in detail processing tasks in the acute state [15,28] and after recovery [11,15,29,30]. Superior detail processing is also found to a degree in first degree relatives (sisters) [11,15,31]. This suggests that a strong focus on detail might be an inherited vulnerability of AN.

Global integration is poor in acute AN, and it may be a consequence of low weight [29]. Therefore, with starvation the balance between global and detail processing which contributes to central coherence is reduced (Figure 1). Moreover, the accentuated set shifting problems in the acute state reduce the flexible switching between the global/detail processing. Thus, weak central coherence may be a secondary consequence of the illness contributing to the maintenance of the illness by impeding adaptation to environmental demands.

#### Clinical formulation relating to obsessive compulsive traits

**Figure 2:**
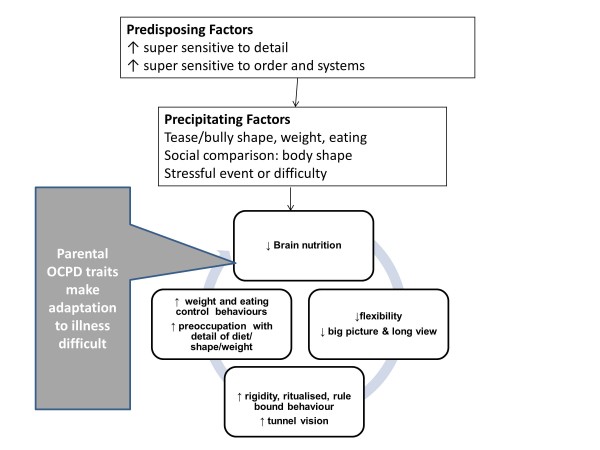
**A diagrammatic formulation of obsessive compulsive personality disorder (OCPD) traits.** Legend. A diagrammatic formulation of obsessive compulsive personality disorder (OCPD) traits showing how they predispose to, and increase, the vulnerability to precipitating factors and also perpetuate the disorder. The grey box indicates how shared familial traits may contribute to the perpetuation of the problem.

A causal and maintenance model derived from the evidence described above is shown in Figure 2. Although people with EDs, in general, have good cognitive abilities with superior attention to detail they show problems in set shifting. Both of these appear to be an inherited traits. These traits in the child make them more susceptible to societal rules and their eye for detail makes aspects of appearance more salient. The traits mean that once dieting behaviour is started (triggered by stress for example), it is undertaken meticulously and the rules and rituals become embedded as rigid habits. Attention to the detail of weight control rules and inflexibility leads to success in the goal of weight loss. This, because of starvation, in turn, impacts on brain function reducing global integration and central coherence and so the focus is further narrowed to the dieting behaviour which become even more habitual. A vicious circle which maintains the disorder develops.

Parents and other family members may share some of these obsessive compulsive traits. These may lead to over controlling parenting (Corfield et al. submitted) which can increase risk particularly in those with the ss form of the serotonin transporter gene [32,33]. Also these traits in parents and other family members may make the process of adjustment and management of the ED behaviours more difficult. A battle for control can ensue as both sides defend their rules, often losing sight of the bigger picture and the longer view of life. This unhelpful reaction inhibits flexible adjustment and serves to maintain ED behaviours.

### Anxiety, avoidance (social and emotional) behaviours

Anxious avoidance of emotions, in particular those aroused by social encounters, insecure attachments, was the second feature within the model considered to be present before the illness. Social avoidance can be a core feature of people with EDs. For example, a personal account of the illness by a medical practitioner described the illness in one word: “isolation” [34]. Difficulties relating to others predate the onset of the illness, can be accentuated by the illness and sometimes persist despite recovery.

People with AN are more likely than controls to report an impoverished social network before the onset of the illness (e.g. having no close friends in childhood; [33,35] fewer social activities [36] and less social support [37]). In part, this relates to temperamental characteristics, as people with EDs are inhibited and shy with internalising problems [38]. Loneliness, feelings of inferiority and high levels of social anxiety [39] are also reported.

In cases ascertained in adolescence, social problems predated the illness in 20% of cases and informed the prognosis, such that cases with social deficits prior to onset at age 15, were found to be impaired 18 years later at follow-up [6,40]. Experiences of teasing, bullying and criticism, often pertaining to weight/shape and eating, are found before the illness [41]. During the illness social networks are often reduced [42,43]. In addition, a sense of inferiority in relationship to others can persist post recovery [44]. Thus, a wide variety of behavioural features support the model, in that an avoidant social phenotype is of relevance to the onset and prognosis. In the next section we examine the form of this social phenotype in more detail. A variety of experimental paradigms have been used to examine factors that may underpin social avoidance in people with EDs, [45] including attention, emotional expression and interpretation and theory of mind.

#### Attentional mechanisms

People with AN differ from healthy controls in that they have an attentional bias towards negative facial expressions such as anger [46-48] criticism [49] and dominance (Cardi et al. submitted) but not towards positive facial expressions such as happiness [46] and compassion [49]. Also, people with AN have been found to direct their gaze less at the face and eyes [50]. These traits remain present, albeit in an attenuated form, in the recovered state and in first degree relatives (twins, parents) (Kanakam et al. submitted Goddard et al. submitted). These findings suggest that there is increased attention to social threat and a decreased attention to social reward and that this may be an inherited vulnerability.

#### Interpretation of social signals

Accurate reading of the intentions and emotions of others is necessary for effective social communication. People with AN have impairments in this domain. A systematic review and meta analysis concluded that people with AN have impairments in recognizing facial emotions, [51]. In contrast, people with BN have little or no impairment in facial emotion recognition [52], and may be better than healthy controls at recognizing negative emotions [53]. In AN, interpreting emotional meaning from the voice [54,55], body movement [56] and from films [54] is also impaired. For the most part, these impairments are less marked after recovery, suggesting that they may be starvation, state effects. There is little evidence that these traits are part of an inherited vulnerability as they are not present in first degree relatives (twins, parents) (Kanakam et al. submitted Goddard et al. submitted).

#### Signaling emotions

The ability to signal one’s own intentions and emotions to others is an important component of effective social communication and people with AN also have impairments in this domain. In a study that examined emotional expression and experience of people with AN, it was found that when watching sad or funny films, people with AN expressed little facial emotional expression and were also less attentive, e.g. turning away more, when watching sad films. This lack of reciprocity and attention contrasts with their self reported emotional reactions which were intense [57]. Also, when people with AN were engaged in a therapeutic emotional skills training video game, they had minimal facial displays of anger and yet on self report measures they had high levels of anger [58]. This suggests that there is inhibited emotional expression and reduced mirroring of emotion. This has not been examined after recovery and in family members.

#### Understanding the mind of another

The most sophisticated aspect of social communication is the ability to understand the mental processes of other people, so called ‘theory of mind’. A proportion of people with AN have problems with theory of mind tasks such as cartoons, which require understanding of implicit thoughts rather than explicit words spoken [59,60]. This has not been examined after recovery and in family members.

A related concept is that of social perception, which denotes “a person’s ability to ascertain social cues from behaviour provided in a social context”, which includes, but is not limited to, emotion cues. Social perception abilities are preserved in ED patients, but those with restricting AN have difficulty in identifying degree of intimacy in social relationships (Renwick et al., submitted).

#### Understanding the self

Finally the perception and understanding of self is an additional component of the social cognition domain that has found to be disturbed in anorexia nervosa. The most widely studied concept in this domain is alexithymia. Sisters of people with anorexia nervosa also have problems in this area which suggests that it might be an additional endophenotype [61]. Abnormalities in other paradigms which measure aspects of interoception such as the rubber hand illusion have also been found [62].

**Figure 3:**
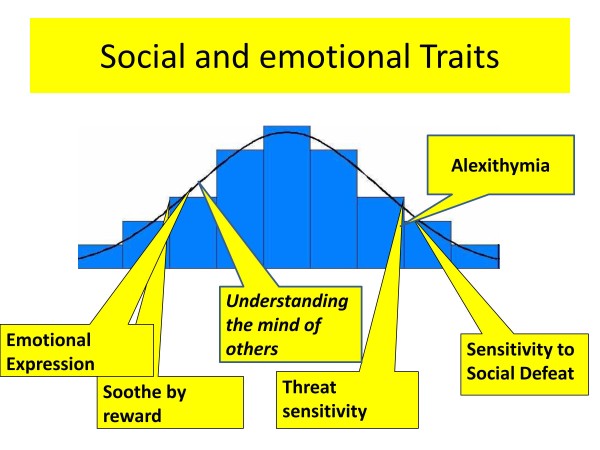
**Social processing traits.** Legend. A diagrammatic representation of social processing traits in eating disorders. Those that are mainly present in the acute starved state are shown in italics.

The traits relating to social processing are displayed in Figure 3.

#### What do we know about the underpinning biology of social processes

Brain activation during social processing in AN differs from that seen in the comparison population. The evoked potential to faces (implicitly and explicitly presented) was reduced both in the acute state and after weight restoration [63]. Two groups have used a task in which moving shapes representing social or non-social/mechanical interactions are shown. Adults (partially recovered) [64] and adolescents (in both the acute phase of AN and after weight restoration (> 100 days treatment)) did not show the expected increase in activation in social processing circuits. On the other hand there was normal activation to emotional faces after recovery AN [65]. Thus, it is possible that some of the anomalies in the brain response to social processing tasks in AN, result from the illness state and are resolved with full recovery but this needs further study.

It is not surprising that social cognition is impaired in the acute state, as this form of cognition requires higher levels of processing. An illustration of this is the social brain hypothesis of Dunbar and colleagues. This theory is based on the observations that throughout the animal and bird kingdoms, social complexity is related to brain size [66,67]. The shrunken, starved brain of AN may not have the functional capacity to optimally participate in the sophisticated communication developed in humans [68]. Moreover, oxytocin functioning, a hormone which is central to aspects of social communication, appears to be disturbed in AN. In the acute state, oxytocin is reduced both peripherally [69] and in the brain [70]. Even after recovery, however, oxytocin released after a test meal is reduced [71].

#### Clinical formulation

**Figure 4:**
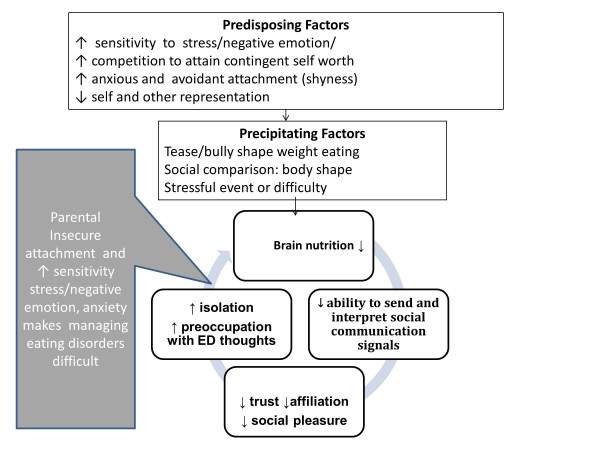
**A diagrammatic formulation of social processing traits.** Legend. A diagrammatic formulation of social processing traits showing how they predispose to and increase the vulnerability to precipitating factors and also perpetuate the disorder. The grey box indicates how shared familial traits may contribute to the perpetuation of the problem.

The balance of evidence is that there are both predisposing inefficiencies in this system and secondary problems acquired as a consequence of the illness. A causal and maintenance model derived from the evidence described above is shown in Figure 4.

Negative self evaluation, internalising strategies and reduced social processing skills predispose to the development of the illness. This is perhaps due to an increased sensitivity to social hierarchies and to negative judgments from others (particularly teasing and criticism about shape, weight or eating) or by the desire to belong and be accepted by attaining social norms (internalisation of thinness). By starving the brain and reducing high level brain function, the symptoms themselves decrease social processing abilities which leads to further withdrawal from social pursuits and poor confidence in situations that require social problem solving abilities [72]. This allows the ED thoughts and behaviours, used to control the biological homeostatic forces, to dominate brain function. The ED becomes the only friend and a vicious circle develops.

There is a small amount of evidence suggesting that some of these traits run in families. This may make social communication in the family more difficult and allow the isolation of AN to take a greater hold.

### Interpersonal relationships

In the initial prototype of the model, the key interpersonal maintaining factors were thought to be high expressed emotion, criticism, hostility and over protection. Systematic reviews of this concept suggest that these behaviours are present and impact on the response to treatment and outcome [73] ( Duclos in press). Accommodating and enabling behaviours [74-78] have been added as behavioural reactions that maintain the illness, another vicious circle.

**Figure 5:**
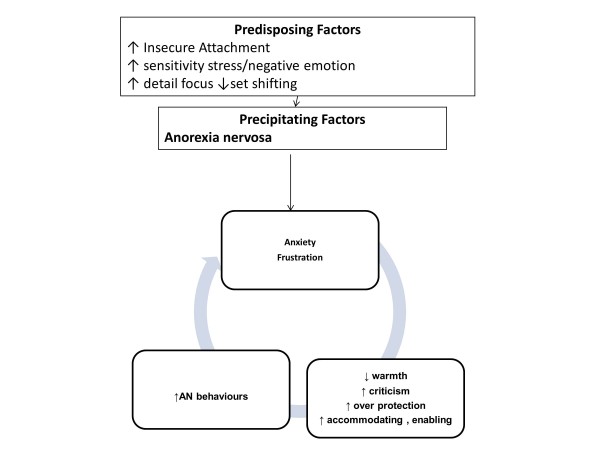
**A diagrammatic formulation of carers involvement within the maintenance of an eating disorder.** Legend. A diagrammatic formulation of how carers own vulnerabilities, insecure attachment, anxiety and OCPD traits predispose to more difficulties coping with the eating disorder leading to anxiety and distress which in turn are associated with high expressed emotion, or accommodation to eating disorder symptoms which act to maintain the disorder.

A formulation for carers showing the interface with eating disorder symptoms is shown in Figure 5. There may be shared OCPD and anxiety traits which become pronounced in the face of the symptoms of anorexia nervosa. This may lead to high expressed emotion (criticism, over protection) and accommodating behaviour which fuels further symptoms.

### Adaptations to the model on the basis of new evidence

The new clinical and behavioural evidence has clarified the model. Although the original key concepts remain, the model has become somewhat more complex as some of the risk factors (strong detail, set shifting and social communication difficulties) appear to be familial vulnerability traits and so have a wider effect by causing a maladaptive response by family members. Also social communication deficits develop or are accentuated as a result of starvation which means that interpersonal relationships become more difficult. Thus, three interlocking formulations relating to cognitive, emotional and interpersonal style may be needed to encapsulate the illness and plan treatment.

### Translational treatment implications

#### Psycho education

This model is a useful heuristic to help patients and their families understand the illness, the role that they can play in causing the illness to persist and how treatment can be managed and directed. Firstly, patients and their families can be helped to understand the many factors that contribute to the illness and which become entwined in a complex manner when the symptoms themselves either alter brain function and so make the individual less capable of change or cause others to react in ways that maybe unhelpful.

Patients and family members may find it helpful to know that the strength in detail, can become problematic when combined with weakened set shifting in the ill state. Also, when patients and families understand how their automatic attention and interpretation mode is biased towards the negative, they can appreciate that their judgments about themselves and the world may be skewed. Furthermore, painful miscommunication can occur because social communication is attenuated by starvation induced, inefficient brain function. Thus their ability to have a balanced appraisal of the emotional state and intentions of themselves and others is weakened.

#### Therapeutic relationship

A common ingredient of many forms of psychotherapy used to treat people with EDs, is the development of a good working alliance and reflective listening to help the individual obtain a more realistic and holistic view of the world and themselves. However, one or three hours a week of therapy is a minimal input compared to the 20 hours a week or more of contact time that most parents/carers have with their offspring [79]. Thus, if families can be helped to develop more effective forms of social communication, this may help them facilitate a restoration of nutritional balance.

In our experience families and therapists find that this conceptualization and explanation of the brain based factors underpinning ED behaviours with an emphasis on the cognitive and social facets of the illness, rather than food alone, can build a more collaborative, inclusive approach. This serves as the rationale to explain why it is helpful to reduce the critical and dominating communication style, which is an understandable reaction to the frustrations of the illness, and instead use a style which elicits a positive self concept by increasing warmth and listening and selectively attending to healthy behaviours rather than anorexia talk. Teaching parents the basic skills of motivational interviewing (MI), such as reflective listening with affirmations and differential attention to “change talk” fosters this communication style. The complex reflections, which are a key part of MI, are a form of theory of mind intervention, in that they seek to encapsulate the gist of the other person’s beliefs motivations and drives. Carers (professional and family members) can model high quality listening and communication. Affirmations serve to allow the individual with an ED to have a positive self image in mind. Improving communication within the family can be generalised to a wider social network. We have found that it is possible for carers to develop high levels of these skills.

#### Specific interventions

We have developed a talking therapy (Maudsley Model of Anorexia Nervosa, MANTRA) based on the cognitive-interpersonal model of AN. This manual-based treatment has been pilot-tested in a case series [80] and a small randomized controlled trial [81]. However, like all forms of talking treatments MANTRA targets complex aspects of brain function which are impaired with a severe and /or chronic form of illness. To improve outcomes further, adjunctive strategies may be needed which use interventions that target some of these brain mechanisms more directly. Examples of these include attention training protocols that have been used to modify threat-related attentional biases in anxiety disorders [82,83] (Renwick et al., in press). The use of pharmacological approaches to supplement psychological interventions for EDs may be of value. For example, oxytocin may enhance the effectiveness of social interventions [84] and promote affiliation and the formation of positive bonding. Finally, neuromodulatory treatments which improve focal brain activation may also have potential [85].

## Conclusion

We have summarised and evaluated the new evidence relating to the cognitive interpersonal model of AN. The key concepts of the model have been clarified and the constructs relating to cognitive, socio-emotional and interpersonal relationships have been more clearly defined, including aspects that pertain to all ED and those that are specific to AN. Primary strengths in detail and vulnerabilities in social and emotional processing may increase the potency of social pressures during adolescence a critical stages of development. These in combination with reduced set shifting allow the illness to take a hold. The secondary consequences of the illness (intra and interpersonal) accentuate these difficulties and cause the illness to persist. There are three sub formulations within the model which can be used to guide treatment. Involving close others, irrespective of individual’s age, is helpful as social and interpersonal reactions are central to the model. New treatments specifically targeted to aspects of the model may improve outcomes.

## Competing interests

The authors declare that they have no competing interests.

## Authors’ contribution

Both authors have contributed to the manuscript and have read and approved the final draft.
